# Functional characterization of a new terpene synthase from *Plectranthus amboinicus*

**DOI:** 10.1371/journal.pone.0235416

**Published:** 2020-07-02

**Authors:** Nur Suhanawati Ashaari, Mohd Hairul Ab. Rahim, Suriana Sabri, Kok Song Lai, Adelene Ai-Lian Song, Raha Abdul Rahim, Wan Muhamad Asrul Nizam Wan Abdullah, Janna Ong Abdullah

**Affiliations:** 1 Department of Cell and Molecular Biology, Faculty of Biotechnology and Biomolecular Sciences, Universiti Putra Malaysia, Selangor, Malaysia; 2 Department of Microbiology, Faculty of Biotechnology and Biomolecular Sciences, Universiti Putra Malaysia, Selangor, Malaysia; 3 Health Sciences Division, Abu Dhabi Women’s College, Higher Colleges of Technology, Abu Dhabi, United Arab of Emirates; Michigan State University, UNITED STATES

## Abstract

*Plectranthus amboinicus* (Lour.) Spreng is an aromatic medicinal herb known for its therapeutic and nutritional properties attributed by the presence of monoterpene and sesquiterpene compounds. Up until now, research on terpenoid biosynthesis has focused on a few mint species with economic importance such as thyme and oregano, yet the terpene synthases responsible for monoterpene production in *P*. *amboinicus* have not been described. Here we report the isolation, heterologous expression and functional characterization of a terpene synthase involved in *P*. *amboinicus* terpenoid biosynthesis. A putative monoterpene synthase gene (*PamTps1*) from *P*. *amboinicus* was isolated with an open reading frame of 1797 bp encoding a predicted protein of 598 amino acids with molecular weight of 69.6 kDa. *Pam*Tps1 shares 60–70% amino acid sequence similarity with other known terpene synthases of Lamiaceae. The *in vitro* enzymatic activity of *Pam*Tps1 demonstrated the conversion of geranyl pyrophosphate and farnesyl pyrophosphate exclusively into linalool and nerolidol, respectively, and thus *Pam*Tps1 was classified as a linalool/nerolidol synthase. *In vivo* activity of *Pam*Tps1 in a recombinant *Escherichia coli* strain revealed production of linalool and nerolidol which correlated with its *in vitro* activity. This outcome validated the multi-substrate usage of this enzyme in producing linalool and nerolidol both in *in vivo* and *in vitro* systems. The transcript level of *PamTps1* was prominent in the leaf during daytime as compared to the stem. Gas chromatography-mass spectrometry (GC-MS) and quantitative real-time PCR analyses showed that maximal linalool level was released during the daytime and lower at night following a diurnal circadian pattern which correlated with the *PamTps1* expression pattern. The *PamTps1* cloned herein provides a molecular basis for the terpenoid biosynthesis in this local herb that could be exploited for valuable production using metabolic engineering in both microbial and plant systems.

## Introduction

*Plectranthus amboinicus* (Lour.) Spreng, locally known as *Bangun-bangun* in Malaysia, is a medicinal herb that belongs in the Lamiaceae family along with the herbs sage, thyme, basil and oregano. It is found naturally throughout the tropics and warm regions of Africa, Asia and Australia [1]. This plant is characterized by its green, succulent, heart-shaped leaves with scalloped edges and can grow to about 50 cm tall with horizontal stems up to 180 cm long [2]. It possesses a distinctive oregano-like odor and flavor that make it an excellent ingredient for culinary purposes. This herb has been traditionally used for treatment of coughs, sore throats, nasal congestion [2], animal and insect bites and also as a breast milk stimulant for hundreds of years [3]. However, the last decade witnessed a large increase of scientific interest in *P*. *amboinicus* research, as evidenced by increasing numbers of publications related to the bioactivities of *P*. *amboinicus*. This plant extract exhibited antibacterial activity against methicillin-resistant *Staphylococus aureus* in a murine model [4], and was proven effective against reproductive tract infections by *Candida albicans*, *Proteus vulgaris* and *Klebsiella pneumoniae* [5]. It has been reported that *P*. *amboinicus* possesses anti-inflammatory and antitumor activities [6], larvicidal [7], and antithrombotic and antioxidant activities [8]. These therapeutic and medicinal properties of *P*. *amboinicus* are mainly attributed to its natural phytochemical compounds present in the essential oils or the plant extract. Essential oils of *P*. *amboinicus* are known to contain high amounts of bioactive compounds, mainly monoterpenoids such as carvacrol, thymol, γ-terpinene, α-terpineol and ρ-cymene with various pharmacological properties [1].

Monoterpenes are C_10_ terpenoids, usually produced by plants, with increasing industrial and clinical applications. In higher plants, terpenoids are synthesized *via* two independent pathways located in separate intracellular compartments. The methylerythritol (MEP) pathway is located in the plastid and responsible for production of mono-(C_10_), di-(C_20_) and tetraterpenes (C_40_), while sesqui-(C_15_), tri-(C_30_) and polyterpenes (C_n_) are synthesized *via* the mevalonate (MVA) pathway in the cytosol. Both pathways produce the universal precursors, isopentenyl pyrophosphate (IPP) and dimethyallyl pyrophosphate (DMAPP), for terpenoids biosynthesis. Further condensation of the two precursors gives rise to linear prenyl pyrophosphate precursors, geranyl pyrophosphate (GPP, C_10_), farnesyl pyrophosphate (FPP, C_15_) and geranyl geranyl pyrophosphate (GGPP, C_20_), and terpene synthases are the key enzymes responsible for catalyzing these substrates into a variety of terpenoids found in plants [9,10]. All terpene synthases have similar properties with respect to their native molecular mass (monomers or homodimers) and requirement for divalent metal ions such as Mg^2+^ and Mn^2+^ for activity. The sequence similarities between terpene synthases are dominated by species relationship regardless of substrate or product specificity, and it was reported that many terpene synthases catalyze formation of multiple products [9,11].

Plants in the Lamiaceae family are known to produce a large variety of terpenoids, and this diversity could be due to expression of multiple terpene synthases and formation of multiple products by individual terpene synthases as previously reported [12–15]. Terpene synthase genes have been previously isolated and characterized from several important Lamiaceae members including *Thymus caespititius* [13,14], *T*. *vulgaris* [16], *Coriandrum sativum* L. [15], *Origanum vulgarae* [12] and *Salvia officinalis* [17]; however the terpene synthases responsible for terpenoids production in *P*. *amboinicus* have not been functionally characterized. Thus, the main objectives of this present study were to isolate and clone the full-length transcript of *P*. *amboinicus* monoterpene synthase, and to functionally express and characterize the recombinant terpene synthase in both *in vivo* and *in vitro* systems. The expression pattern of the isolated transcript that was involved in the formation and emission of terpenoids in different plant parts under day/night period was also explained. The information obtained from this study will benefit future exploitations of the isolated enzyme for terpenes biosynthesis in a simpler heterologous microbial or plant system.

## Materials and methods

### Plant material

The *P*. *amboinicus* leaves were collected from plants grown at the Faculty of Biotechnology and Biomolecular Sciences, Universiti Putra Malaysia, Selangor, Malaysia. The plant was identified and authenticated taxonomically at the School of Environmental Science and Natural Resources, Universiti Kebangsaan Malaysia, Selangor, Malaysia. The voucher specimen, UKMB40411, was deposited in the herbarium facility. Fresh plant samples were used directly for GC-MS analysis of the volatile compounds. For RNA extraction, the leaves were harvested and immediately frozen in liquid nitrogen and kept at– 80°C until ready for the extraction process.

### GC-MS analysis of *Plectranthus amboinicus* volatiles

The volatiles released from the *P*. *amboinicus* leaves and stems harvested at 2.00 AM, 8.00 AM, 2.00 PM and 8.00 PM were collected using headspace solid phase microextraction (HS-SPME) equipped with a 100 μm polydimethylosiloxane (PDMS) fiber (Supelco) and analyzed using an Agilent 7890A gas chromatograph coupled to an Agilent 5975C quadrupole mass detector (Agilent Technologies, Santa Clara, USA). The instrument was equipped with an Agilent HP-5MS capillary column (30 m x 250 μm inner diameter x 0.25 μm film) and helium was used as the carrier gas at a flow rate of 1 ml per min. The SPME fiber was conditioned at the GC injection port at 250°C for 5 min before use. Approximately 1 g of the *P*. *amboinicus* tissue was placed in a 20 ml headspace vial fitted with a silicone septum screw cap. Following 10 min of sample conditioning at room temperature, the SPME fiber was exposed to the headspace for 30 min at 60°C and immediately desorbed in the gas chromatograph injector at 250°C for 15 min using a splitless mode. The GC oven was maintained at 40°C for 2 min, gradually increased to 175°C at a rate of 5°C/min and then an increment up to 250°C at 90°C/min. Linear retention index (LRI) was determined through the injection of a C8 to C20 series of straight chain n-alkanes (Sigma Aldrich, USA) and calculated in accordance to van Den Dool and Kratz [18]. The volatile compounds were identified by mass spectra comparison using a MSD Chemstation Enhanced Data Analysis Software (E.02.02.1431 version, Agilent Technologies) and the National Institute of Standards and Technology library database (NIST 14). In addition, the compounds were tentatively identified by comparing the experimental retention indices with the theoretical ones obtained from the literatures. The relative amount of the individual component was expressed as a percentage of the peak area of respective compound over the total peak areas of all identified volatiles.

### Isolation of full-length *P*. *amboinicus* monoterpene synthase gene (*PamTps1)*

Based on the *P*. *amboinicus* transcriptome library (SRA Accession No.:SRR7842030) previously created from matured leaves and sequenced using the MiSeq Illumina platform (Mohd Hairul Ab. Rahim, unpublished), a 1361 bp partial terpene synthase transcript (Accession No.: GGXS01005129) that showed the highest sequence similarity to known plant monoterpene synthases was selected and designated as *PamTps1*. Total RNA was extracted using Tri Reagent (MRC, USA) following the manufacturer’s protocol. The RNA was quantified and its purity was determined using UV-VIS spectrophotometer (NanoDrop 1000, Thermo Scientific, USA), and its integrity was assessed on a 1.2% (w/v) agarose gel. Rapid Amplification of cDNA Ends (RACE) was conducted using SMARTer RACE Kit according to the manufacturer’s protocol with minor modifications (Clontech, USA). For the first strand cDNA synthesis, 1 μg of total RNA was reverse transcribed using SMARTScribe Reverse Transcriptase (Clontech, USA) according to the manufacturer’s instructions. The components of the RACE reactions were 1X Advantage 2 PCR buffer, 0.8 mM dNTP mix, 1 μM gene-specific primer (5’- CCCTATCCCTCACAAATGGGAGTTTCT-3’), 1X Universal Primer A Mix, 2.5 μl of 5’-RACE-Ready cDNA, 1X Advantage 2 Polymerase Mix and PCR-grade water added to a final volume of 50 μl. The gene-specific primer was designed at the conserved region of the partial sequence obtained from the transcriptomic data (SRA Accession No.:SRR7842030 and Accession No.: GGXS01005129) that fulfilled the requirements of 23–28 bp and 50–70% GC contents with a melting temperature ranging from 65 to 72°C. The gene-specific primer was synthesized by Bioneer, Korea. The RACE amplification was conducted using a touch-down program set at 5 cycles of 94°C for 30 sec, 72°C for 3 min, 5 cycles of 94°C for 30 sec, 70°C for 30 sec, 72°C for 3 min, and 25 cycles of 94°C for 30 sec, 68°C for 30 sec, 72°C for 3 min, and a final extension of 72°C for 10 min. The amplified product was cloned into pGEMT-Easy vector (Promega) and sent for sequencing (Bioneer, Korea). Based on the assembled fragments obtained by RACE and the known partial sequence, the full-length cDNA was then amplified in a PCR reaction containing 1X *Pfx* buffer, 0.3 mM dNTP mixture, 2 mM MgSO_4_, 0.5 U of Platinum™ Pfx DNA Polymerase (Thermo Fisher Scientific, USA), 0.8 μM of *PamTps1*-F (5’-CAACGCAGAGTACATGGGATGGAGCAA-3’) and *PamTps1*-R (5’-GCATTTGTTCAGACATATGGATGGAACAGC-3’) primers, and 2.5 μl of 5’-RACE-Ready cDNA. Amplification was done at 94°C for 2 min, followed by 35 cycles of 94°C for 15 sec, 68°C for 1 min, 68°C for 3 min, and a 10 min final extension at 68°C. The successful amplicon was cloned into a pGEMT-Easy vector (Promega) and sent for sequencing (Bioneer, Korea).

### Full-length sequence analysis and phylogenetic tree construction

The full-length transcript of *Pam*Tps1 was aligned against the non-redundant protein database via the BLASTx algorithm. The N-terminal signal peptide sequence was predicted using ChloroP 1.1 Server (http://www.cbs.dtu.dk/services/ChloroP/) [19] and TargetP 1.1 Server (http://www.cbs.dtu.dk/services/TargetP/) [20]. The presence of conserved motifs shared by all known terpene synthases were identified by protein sequence alignments between *Pam*Tps1 and linalool synthase of *Perilla frutescens* var. *hirtella* (ACN42013.2), *P*. *citriodora* (AAX16075.1) and *Lavandula latifolia* (ABD77417.1), *S*. *rosmarinus* pinene synthase (ABP01684.1), *P*. *setoyensis* geraniol synthase (ACN42010.1), γ-terpinene synthase of *T*. *caespititius* (AID51195.1), *T*. *serpyllum* (AGT29345.1) and *O*. *syriacum* (AEO27879.1), *L*. x *intermedia* 3-carene synthase (ARA91313.1), *T*. *caespititius* α-terpineol synthase (AGK88250.1) and *O*. *vulgare* terpene synthase 5 (ADK73617.1) using Clustal Omega (https://www.ebi.ac.uk/Tools/msa/clustalo/) [21] and BoxShade version 3.21 server (https://embnet.vital-it.ch/software/BOX_form.html). The evolutionary relationships of *Pam*Tps1 were inferred using the Neighbor-Joining method and the bootstrap consensus tree was inferred with 1000 replicates computed via the PHYLogeny Inference Package version 3.695 (PHYLIP) [22].

### Functional expression of *Pam*Tps1

The ORF of *PamTps1* excluding the N-terminal transit peptide was amplified using *PamTps1* forward (5’-ATTC*AAGCTT*ATGAAGCCCGCTGTTGAAGCC-3’) and reverse (5’-ATTC*CTCGAG*TCCAGAGCCGACATATGGATGGAACAG-3’) primers with restriction enzyme (RE) sites shown in italics to create *Hin*dIII and *Xho*I (New England Biolabs, Canada) overhangs for use during ligation into a pET32b(+) bacterial expression vector (Merck, Germany), respectively. The three amino acids spacer of GSG (underlined) was also incorporated into the reverse primer. The PCR components and programs were as previously described with an annealing temperature of 64°C. The successful amplicon was cloned into the pET32b(+) expression vector (Merck Millipore, USA) and transformed into *E*. *coli* Rosetta™ 2 (DE3) competent cells (Merck Millipore, USA), and presence of the insert was confirmed using RE digestions, followed by sequencing.

A single colony of recombinant expression cells harboring pET32b:*PamTps1* and empty vector pET32b, respectively, was inoculated into 10 ml Luria-Bertani (LB) medium containing 50 μg/ml carbenicillin and 34 μg/ml of chloramphenicol and grown at 37°C, 250 rpm overnight. Approximately 5 ml of the overnight culture was transferred into 95 ml of fresh LB medium supplemented with the same antibiotics and grown at 37°C until OD_600nm_ ~ 0.6–0.8 was reached. Protein expression was induced by the addition of isopropyl-β-D-thiogalactopyranoside (IPTG) to a final concentration of 0.5 mM and incubated with shaking at 150 rpm, 28°Cfor 16 h. After induction, the cells were harvested by centrifugation at 3214 x *g*, 4°C for 30 min and kept frozen at -20°C until ready for protein extraction. Protein extraction was conducted using the BugBuster Protein Extraction Reagent (Novagen, USA) according to the manufacturer’s instructions. The lysate was then centrifuged at 12,633 x *g*, 4°C for 20 min to obtain the soluble protein fraction while the insoluble pellet was treated with 6 M urea at 4°C overnight followed by centrifugation to recover the inclusion body (IB) fraction. Buffer exchange to assay buffer containing 10 mM Tris-Cl, 10% (v/v) glycerol, 1 mM dithiothreitol (DTT) and 10 mM MgCl_2_ at pH 7.5 [14] was conducted using Amicon® Centrifugal Filter (MW cutoff = 30 kDa) (Merck Millipore, USA) following the manufacturer’s instructions.

The soluble protein fraction containing the His-tagged protein was purified using a HisTrap™ HP 5 ml column (GE Healthcare, USA) according to the manufacturer’s instructions. The eluted fractions were pooled and concentrated to 1 ml using Amicon® Centrifugal Filter and further purified by gel filtration chromatography using Superdex 200 10/300 GL column (GE Healthcare, USA). The fractions containing pure *Pam*Tps1 were eluted isocratically using the assay buffer at a flow rate of 0.5 ml/min.

The purified recombinant *Pam*Tps1 including the soluble and insoluble protein extracts were resolved on 10% sodium dodecyl sulfate polyacrylamide gel electrophoresis (SDS-PAGE) and visualized by Coomassie blue staining. Western blotting for detection of the His-tagged protein was also conducted using anti-polyHistidine-HRP antibody (Sigma-Aldrich, USA) (dilution 1∶2000) and detected using the SuperSignal® West Pico Chemiluminescent HRP substrate (Pierce, USA) following the manufacturer’s instructions.

### Functional characterization of *Pam*Tps1

Enzyme assays were performed accordingly in a glass vial using 25 μg recombinant protein in 100 μl reaction buffer containing 10 mM Tris-Cl, 10% (v/v) glycerol, 1 mM DTT and 10 mM MgCl_2_ [14]. Phosphatase inhibitors of sodium tungstate (Na_2_WO_4_) and sodium fluoride (NaF) were added at final concentrations of 0.1 mM and 0.05 mM, respectively, to prevent geraniol formation. The enzymatic reaction was initiated by addition of 20 μM GPP or FPP (Sigma Aldrich, USA) and incubated at 30°C with constant shaking for 2 h. Terpene products were collected by using a SPME fiber exposed for 30 min in the headspace above the assay mixture at 60°C in a water bath and analyzed by GC-MS. The oven temperature was programmed at 50°C and gradually increased to 280°C at a rate of 10°C/min for 3 min. The temperature of the ion source and transfer line were set at 220°C and 280°C, respectively, and the electron impact mass spectra were recorded at 70 eV ionization energy. The identities of the terpene compounds were determined with referral to the NIST 14 library, as well as comparison of mass spectra and retention times with authentic standards (Sigma-Aldrich, USA). The *in vivo* activity of *Pam*Tps1 was conducted using the recombinant *E*. *coli* strain harboring the expression construct pET32b:*PamTps1* grown under the conditions as described in the protein expression section and the *E*. *coli* host harboring an empty vector was used as a control. Cultures were sampled at 24 h of post-induction and the volatile terpenoid compounds in the headspace of each culture were analyzed using HS-SPME-GC-MS as described in the assay reactions.

### Gene expression analysis of *PamTps1*

For the expression analysis of *PamTps1* in various tissues, leaf and stem samples were collected at 8.00 AM, 2.00 PM, 8.00 PM and 2.00 AM with three biological replicates. The plant samples were immediately frozen and stored at—80°C until the extraction process using the Tri Reagent (MRC, USA). Total RNA of 1 μg was treated for genomic elimination and reverse transcribed using QuantiTect® Reverse Transcription kit (Qiagen, USA) according to the manufacturer’s protocol. Quantitative RT-PCR was conducted to determine *PamTps1* transcript abundance involved in the production of linalool and nerolidol. A 291 bp fragment in the 3’ region of *PamTps1* was amplified using the gene-specific primers (S1 Table). The experiments were performed using QuantiNova SYBR Green PCR Master Mix (Qiagen, USA) as per manufacturer’s procedure and the real-time cycler program as follows: 95°C for 2 min, 40 cycles of 95°C for 5 s, 60°C for 10 s, followed by a melting curve analysis of 65–95°C with 0.5°C increments. Sequencing analysis was performed to verify amplification of the expected region of *PamTps1*. Each primers pair was validated using a standard curve of serial cDNA dilutions to calculate the correlation coefficient and amplification efficiency. Three reference genes namely elongation factor G *(EF-G)*, tubulin and adenine phosphoribosyl transferase *(APRT)* were used for normalization of the qPCR data. The primers for the qPCR analysis are listed in the S1 Table.

## Results and discussion

### Volatiles profiling of *Plectranthus amboinicus* leaves

**Fig 1:**
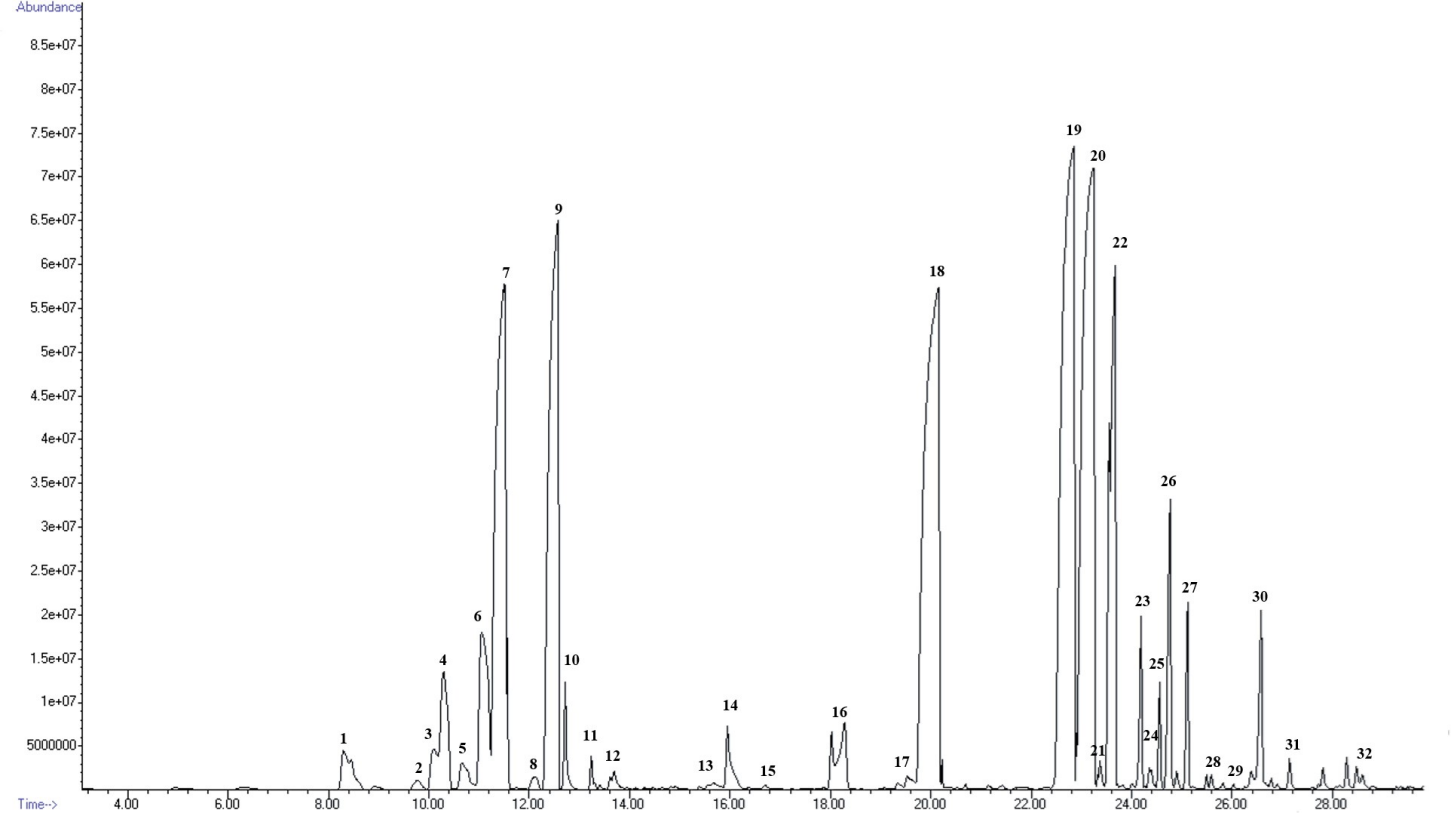
Total ion chromatogram of *Plectranthus amboinicus* leaf volatiles harvested at 8.00 AM extracted using headspace solid phase microextraction (HS-SPME). The numbers corresponded to the compounds detected as described in Table 1.

Headspace—solid phase microextraction—gas chromatography—mass spectrometry (HS-SPME-GC-MS) analysis demonstrated that the *P*. *amboinicus* volatiles were dominated by α-bergamotene (19.5%), carvacrol (19.6%), caryophyllene (19.2%), p-cymene (8.2%), γ-terpinene (10.5%) and humulene (5.6%) (Table 1, Fig 1). Seven terpene alcohols including 1-octen-3-ol, terpinene-4-ol, linalool and nerolidol were detected which formed 2.35% of *P*. *amboinicus* volatiles. Carvacrol has a characteristic pungent spicy-woody odor which contributes to the strong oregano-like aroma [23], and together with the high relative percentages of citrus odor of α-bergamotene [24] and caryophyllene (spicy-woody) [25] collectively contribute to the unique, strong aromatic odor of *P*. *amboinicus*. This result is in agreement with the known volatile constituents of *P*. *amboinicus* previously reviewed by Arumugam et al. [1]. However, the phytochemical composition of plants may vary depending on geographical locations, climatic conditions, methods of extraction and identification. The volatile constituents of *P*. *amboinicus* leaves collected from Uganda which were also extracted using HS-SPME showed the presence of linalool (50.3%) as the main component with other detected volatiles such as carvacrol (14.3 4%), geranyl acetate (11.7%), nerol acetate (11.6%) and γ-terpinene (3.2%) [26]. On the other hand, *P*. *amboinicus* oil from Cambodia was shown to contain dominant constituents of thymol (57.4%), carvacrol (13.5%), γ-terpinene (5.6%) and p-cymene (5.2%) [27]. Interestingly, thymoquinone and thymohydroquinone, phytochemical compounds with potential application as an anti-cancer drug that can be found in *Nigella sativa* were also detected in *P*. *amboinicus* albeit in small amounts. The presence of thymoquinone in *P*. *amboinicus* has also been documented by Chen et al. [28] in water-hexane extract with further identification using mass spectrophotometer and NMR analysis.

**Table 1:** Compounds identified from *Plectranthus amboinicus* leaf volatiles harvested at 8.00 AM using HS-SPME-GC-MS analysis.

No	RT^a^ (min)	Compounds	RI^b^	Relative content^c^ (%)
1	8.304	β-Thujene	924	0.432 ± 0.048
2	10.081	1-Octen-3-ol	983	1.140 ± 0.054
3	10.279	β-Myrcene	990	1.372 ± 0.147
4	10.653	α-Phellandrene	1002	0.458 ± 0.030
5	11.043	α-Terpinene	1015	2.508 ± 0.212
6	11.434	ρ-Cymene	1027	8.208 ± 0.445
7	12.069	β-Ocimene	1048	0.149 ± 0.015
8	12.512	γ-Terpinene	1063	10.475 ± 0.465
9	13.229	Terpinolene	1086	0.161 ± 0.003
10	13.683	Linalool	1101	0.163 ± 0.020
11	15.665	Isoborneol	1169	0.131 ± 0.012
12	15.951	(-)-Terpinen-4-ol	1178	0.821 ± 0.026
13	16.370	α-Terpineol	1193	0.021 ± 0.010
14	18.032	Thymoquinone	1252	2.296 ± 0.753
15	19.343	Thymol	1294	0.331 ± 0.013
16	20.171	Carvacrol	1326	19.583 ± 1.937
17	21.144	Eugenol	1363	0.042 ± 0.004
18	22.794	Caryophyllene	1430	19.211 ± 2.355
19	23.213	α-Bergamotene	1447	19.529 ± 1.255
20	23.347	(+)-Epi-β-Santalene	1453	0.201 ± 0.014
21	23.627	Humulene	1464	5.636 ± 0.623
22	24.181	(E)-β-Famesene	1486	1.014 ± 0.056
23	24.385	(-)-Zingiberene	1501	0.181 ± 0.068
24	24.548	α-Muurolene	1508	0.478 ± 0.077
25	24.758	β-Bisabolene	1517	2.195 ± 0.079
26	25.108	β-Sesquiphellandrene	1532	1.080 ± 0.039
27	26.034	(±)-*trans*-Nerolidol	1571	0.033 ± 0.008
28	26.384	Thymohydroquinone	1586	0.213 ± 0.056
29	26.576	Caryophyllene oxide	1594	1.419 ± 0.123
30	27.148	Humulene epoxide II	1615	0.190 ± 0.022
31	28.797	α-Bisabolol	1688	0.039 ± 0.010

^a^ Retention time (RT) in min

^b^ van den Dool and Kratz retention index calculated for HP-5MS column

^c^ Relative peak area expressed as percentage of the peak area of corresponding compound over the total peak areas of all identified volatiles.

### Isolation of full-length monoterpene synthase gene and sequence characterization

The partial *PamTps1* transcript (Accession No.: GGXS01005129) was identified from the *P*. *amboinicus* transcriptome (SRA Accession No.:SRR7842030) using the BLAST alignment that showed hits to known terpene synthases and also on the basis of the presence of its conserved sequence characteristics that are shared by all terpene synthases. The full-length *PamTps1* transcript (Accession no: MK050501) contained an open reading frame (ORF) of 1797 bp encoding a protein of 598 amino acids with theoretical isoelectric point (pI) and molecular weight of 5.40 and 69.6 kDa, respectively. The transcript was predicted to contain a 52 amino acid N-terminal chloroplast transit peptide, which most likely targets this protein to the plastid, the location of monoterpenes biosynthesis found in other Lamiaceae members. This predicted transit peptide was characterized by high contents of serine and threonine, and low number of acidic residues which in accordance to Williams et al. [29] is expected for the *Tpsb* subfamily.

The sequence identity of *Pam*Tps1 at the amino acid level was compared via BLASTp algorithm against the NCBI non-redundant protein database and showed 60–70% amino acid sequence identity to monoterpene synthases of other Lamiaceae members including *T*. *caespititius* γ-terpinene synthase (AID51201.1) [14], *S*. *rosmarinus* pinene synthase (ABP01684.1), *P*. *frutescens* linalool synthase (AAL38029.1) and *P*. *setovensis* geraniol synthase (ACN42010.1) [30]. This analysis confirmed that the sequence similarity among Lamiaceae terpene synthases was relatively high but may not necessarily be linked to the catalytic function of the enzyme. Based on the sequence similarity and presence of N-terminal transit peptide, *Pam*Tps1 was designated as a putative monoterpene synthase.

**Fig 2:**
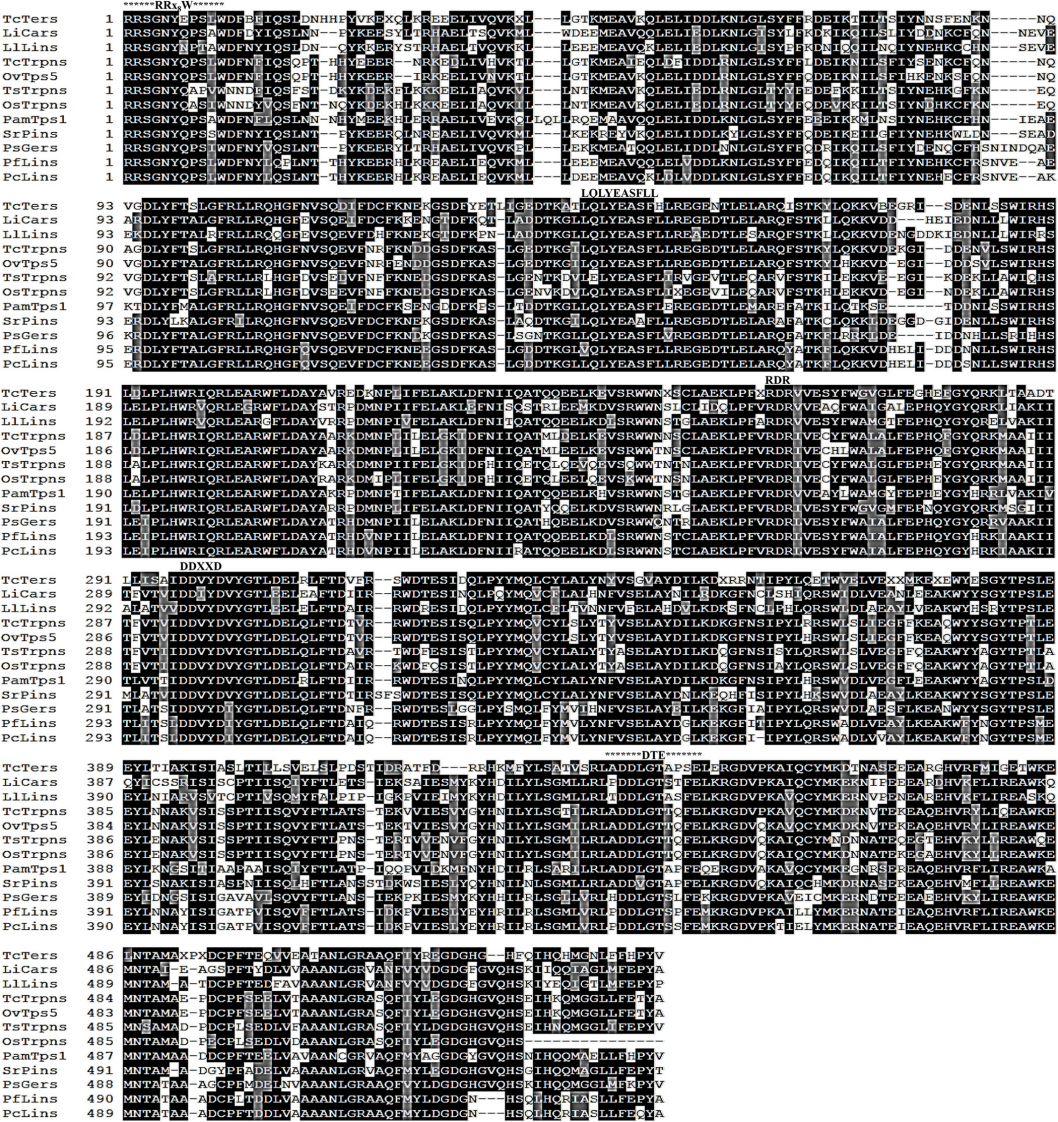
Alignment of *Pam*Tps1 amino acid sequence without transit peptide with other plant monoterpene synthases using Clustal Omega and BoxShade server. Conserved domains of RRx8W, LQLYEASFL, DDxxD, GTLxEL and DTE were labelled. AGK88250.1: *T*. *caespititius* α-terpineol synthase; ARA91313.1: *L*.x *intermedia* 3-carene synthase; ABD77417.1: *L*. *latifolia* linalool synthase; AID51195.1: *T*. *caespititius* γ-terpinene synthase; ADK73617.1: *O*. *vulgare* terpene synthase 5; AGT29345.1: *T*. *serpyllum* γ-terpinene synthase; AEO27879.1: *O*. *syriacum* γ-terpinene synthase; ABP01684.1: *S*. *rosmarinus* pinene synthase; ACN42010.1: *P*. *setoyensis* geraniol synthase; ACN42013.2: *P*. *frutescens* var. *hirtella* linalool synthase and AAX16075.1: *P*. *citriodora* linalool synthase.

Despite the sequence diversity, terpene synthases share several conserved amino acid residues both at the N- and C-terminal protein domains. These conserved motifs were found in the deduced amino acid sequence of *Pam*Tps1 (Fig 2) notably the tandem arginine motif (RRx_8_W) located downstream of the transit peptide at the N-terminal region of monoterpene synthases. Deletion of this motif on the *Mentha spicata* limonene synthase was found to affect the ability of the enzyme to utilize geranyl pyrophosphate as a substrate, suggesting that this motif might be involved in the isomerization of geranyl pyrophosphate to a cyclizable intermediate [29]. This arginine pair has also been reported to stabilize the closed active site in the enzyme-ligand complexes reaction [31]. The LQLYEASFLL motif which was also found in *Pam*Tps1 but as a LQLYEASFLE sequence is another conserved motif that is believed to be part of the active site and might be involved in substrate binding [15,32]. Another conserved region in terpene synthases is the aspartate-rich DDxxD motif that has been found in almost all isolated plant terpene synthases. This highly conserved motif, which occurs as DDVYD in *P*. *amboinicus*, is located at the C-terminal domain of all terpene synthases and is known to be involved in the binding of divalent metal ion cofactor such as Mg^2+^ or Mn^2+^ to initiate binding and activation of the diphosphate moiety of the substrate [33–35]. The DDxxD motif located at the entrance of the catalytic site was demonstrated to be critical in positioning the substrate for catalysis as mutation of this motif often led to decreased catalytic activity and abnormal product [36,37]. Besides that, an additional metal cofactor binding motif NSE/DTE which evolved from a second aspartate-rich region to form a consensus sequence of (L,V)(V,L,A)(N,D)D(L,I,V)x(S,T)xxxE occurred as LADDLGTAPFE in *Pam*Tps1. Both the DDxxD and NSE/DTE motifs bind to a trinuclear magnesium ions cluster and are involved in the fixation of pyrophosphate substrate. *Pam*Tps1 also contains other motifs such as RxR and GTLxEL that are postulated to be part of terpene synthases active site [17,38] and occur as RDR and GTLDEL, located about 35 amino acids upstream and two amino acids downstream of DDxxD, respectively.

### Functional expression of *Pam*Tps1

The N-terminal transit peptide necessary for plastidial targeting of monoterpene synthases has been reported to facilitate formation of inclusion bodies in an *E*. *coli* expression system. High yield expression of soluble monoterpene synthases can be accomplished by truncation of the transit peptide from the coding region to remove the target sequence [11]. Previous studies on bacterial expression of terpene synthases isolated from other members of Lamiaceae such as *M*. *spicata* limonene synthase and *T*. *caespititius* γ-terpinene synthase demonstrated the expression of functional enzymes upon removal of the transit peptide upstream of the double arginine motif [13,29]. Therefore, the signal peptide was removed from *PamTps1* sequence during cloning in order to achieve expression of a soluble and functional putative monoterpene synthase in an *E*. *coli* system. The complete *PamTps1* ORF was 1797 bp, of which 147 bp corresponding to the putative transit peptide was removed to improve protein solubility during expression. The truncated *PamTps1* was cloned into pET32b(+) expression vector that contained a dual-fusion tag consisting of thioredoxin (TrxA) and histidine (His_6_) to give a predicted mass of 79 kDa for the fusion protein. The truncated *PamTps1* was expressed in Rosetta™ 2(DE3) strain which supplied rare tRNAs to cope with the differences of codon usage between *PamTps1* and *E*. *coli* that may impede translation due to the demands for tRNAs that may be lacking in the bacterial host. Protein expression analysis for the clone harboring the truncated *PamTps1* was compared with the control expression containing the empty pET32b(+) vector. The recombinant protein was then purified using immobilized metal affinity chromatography (IMAC) followed by gel filtration chromatography to yield a pure protein for functional characterization study. The SDS-PAGE and Western blot analyses showed the presence of a corresponding protein band of an estimated size of ~79 kDa (S1 Fig).

### Functional characterization of *Pam*Tps1

**Fig 3:**
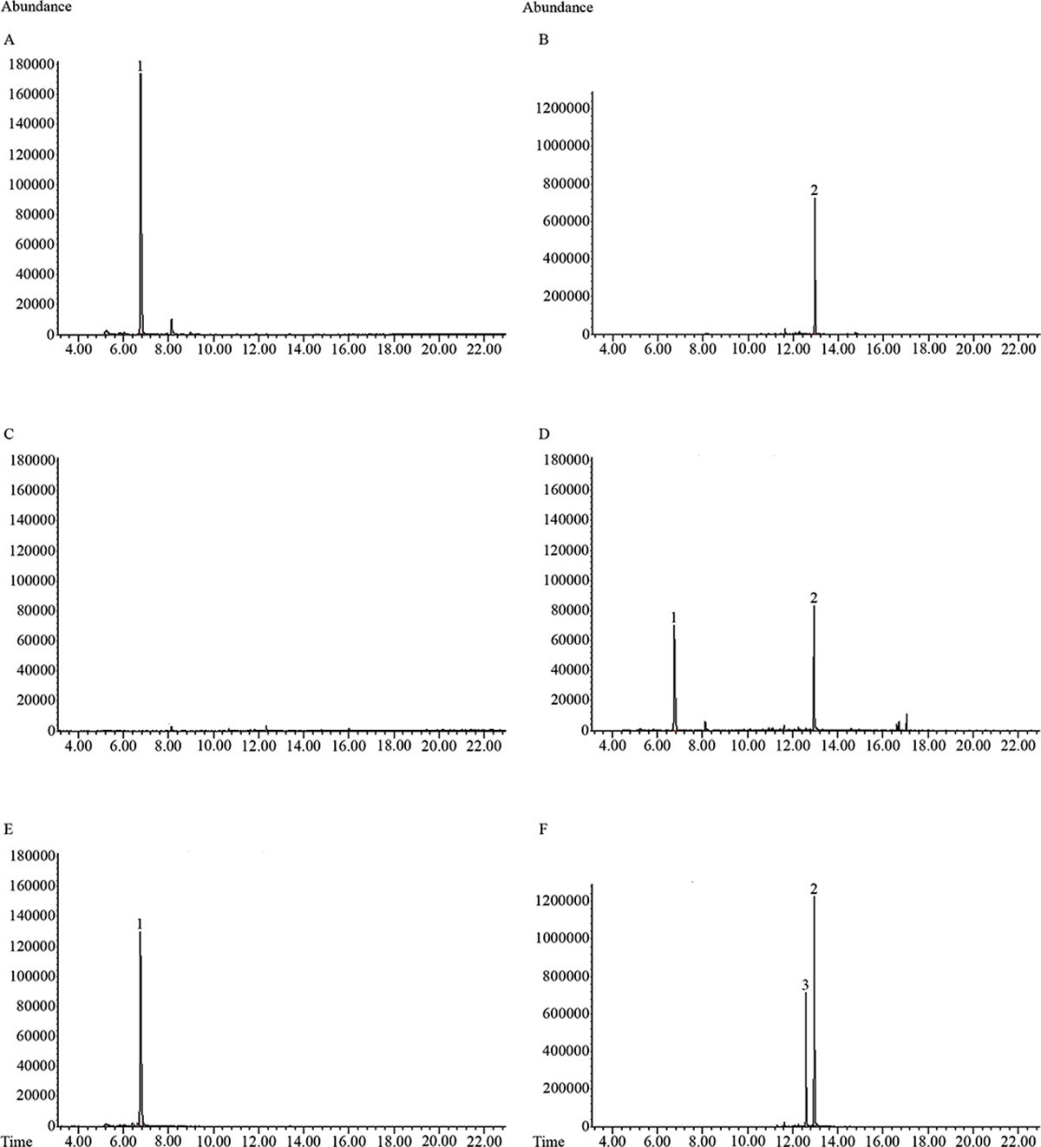
GC-MS chromatograms (selected ion, m/z = 93) of *Pam*Tps1 products generated both in *in vitro* and *in vivo* systems. *Pam*Tps1 enzymatic reaction incubated with (A) GPP or (B) FPP; products generated in *E*. *coli* harboring (C) empty vector and (D) *PamTps1*; (E) (-)-linalool standard and (F) *cis*- and *trans*-nerolidol standards. *In vitro Pam*Tps1 reactions produced exclusively linalool and nerolidol when incubated with GPP and FPP, respectively. In recombinant *E*. *coli*, *PamTps1* produced both linalool and nerolidol. Only sample peaks higher than the negative control were marked with numbers. Corresponding compounds are: 1 = linalool (retention time = 6.7 min); 2 = *trans*-nerolidol (retention time = 13.0 min) and 3 = *cis*–nerolidol (retention time = 12.6 min).

Most of the previously described terpene synthases were multi-product enzymes where the identity and relative abundance of the terpene products did not accurately predict the protein function [15,39,40]. Thus, the activity of *Pam*Tps1 was investigated through *in vitro* enzymatic assay and *in vivo* expression in an *E*. *coli* system. The *Pam*Tps1 enzymatic reaction demonstrated that this protein predominantly catalyzed formation of linalool from GPP confirming that *Pam*Tps1 is a functional monoterpene synthase (Fig 3A). Interestingly, when FPP was provided as a substrate, *Pam*Tps1 was able to synthesize sesquiterpene nerolidol, the C_15_ analogue of linalool (Fig 3B). The identification of linalool and nerolidol produced from *Pam*Tps1 enzymatic reaction was conducted by comparing their retention times and mass spectra with the authentic standard materials and NIST14 library (Fig 3 and S2 Fig). Smaller peaks of linalool and nerolidol were observed in the control reactions without enzyme which could arise as a result of non-enzymatic solvolysis of GPP and FPP in the presence of metal ions (S3 Fig) [41]. However, it was evident from the chromatogram that the significant and higher signal abundances of linalool and nerolidol as compared to the control reactions were the products of *Pam*Tps1 enzymatic reactions, albeit with partial contamination of solvolysed products of the substrates. These enzymatic products of *Pam*Tps1 were also detected in the volatiles composition of *P*. *amboinicus* although only present at 0.2% of the total volatiles detected (Table 1).

Some terpene synthases may behave differently in *in vivo* as compared with the *in vitro* due to differences in cofactors availability and other biochemical conditions [42,43]. It was previously shown that terpene synthase products can vary depending on the expression host and the subcellular localization of the protein [42,44,45]. Thus, in order to demonstrate whether multi-substrate activity of *Pam*Tps1 extends to the *in vivo* environment, microbial production of terpenoids in the *E*. *coli* system was conducted. The terpenoid profile produced in the recombinant *E*. *coli* host up to 24 h of post-induction was illustrated in Fig 3F. It was observed that the recombinant *E*. *coli* harboring *PamTps1* was capable of producing both linalool and nerolidol *in vivo* without supplementation of any exogenous GPP or FPP substrates, which correlated with the *in vitro* activity of *Pam*Tps1. This outcome validated the multi-substrate use of this enzyme in producing linalool and nerolidol in both the *in vivo* and *in vitro* systems. Hence, we designated *Pam*Tps1 as a linalool/nerolidol synthase, and this is the first report describing isolation and functional characterization of such substrate promiscuity of a terpene synthase from *P*. *amboinicus*. Recent advances and progresses in the characterization of enzymes responsible for terpenoids biosynthesis in plants and bacteria have revealed the existence of multi-substrate terpene synthases capable of synthesizing terpenes of different chain lengths depending on corresponding substrate availability. Pazouki and Niinemets [46] highlighted that there were at least 40 confirmed cases of multi-substrate enzymes among plant terpenoids, suggesting that the substrate promiscuous terpene synthases are prevalent within the plant kingdom.

Based on the functional assay study, it was evident that *Pam*Tps1 is a high-fidelity, multi-substrate enzyme capable of performing both monoterpene and sesquiterpene activities in *in vitro* and *in vivo* systems for the formation of linalool and nerolidol. In *planta*, the formation of linalool and nerolidol requires either two specialized terpene synthases (linalool and nerolidol synthases) or a substrate promiscuous linalool/nerolidol synthase. Linalool synthases are widely identified from both angiosperms and gymnosperms, and are considered a defective monoterpene cyclase that can catalyze the initial ionization and rearrangement of GPP but cannot proceed to the subsequent cyclization steps [47]. It was an interesting observation that most papers reported linalool synthase or nerolidol synthase or linalool/nerolidol synthase showed high-fidelity activity by producing exclusively either linalool or nerolidol instead of multiple compounds as demonstrated by the promiscuous terpene synthases [33,48–52]. The *Pam*Tps1 shared a similarity of less than 36% with other linalool/nerolidol synthase from *V*. *vinifera* [53,54], *Streptomyces clavuligerus* [55], *A*. *majus* [48] and *S*. *lycopersicum*. Sequence comparison of *Pam*Tps1 with plant monoterpene synthases showed that Lamiaceae linalool synthases differ from others by a three-amino acid deletion (i.e. between residues 524–526 in *Pam*Tps1) at the C-terminal region of the protein (Fig 2) which structurally provided more water access to the active site of linalool synthase and caused premature quenching by water capture [47]. However, no deletion of such amino acids was observed in *Pam*Tps1, and it was postulated that this premature quenching was due to the less efficient active site closure in the enzyme-ligand complexes which thereby was unable to shield reactive carbocation intermediates from the water molecule [56]. It will be interesting to further explore this *Pam*Tps1 to identify the structural features that confer the multi-substrate function that can be exploited to engineer terpene synthases with high fidelity and specificity for production of terpenoids in a microbial cell factory.

### Expressional analysis of *PamTps1*

Volatile terpenoids are often synthesized and emitted from specific plant tissue at a particular time that correlates with the spatio-temporal expression of their terpene synthases, suggesting that the terpenoid biosynthesis is transcriptionally regulated [57]. The leaf and stem tissues of aromatic plants were generally associated with the presence of secretory structures that produced large quantities of volatile terpenoids consisting mostly monoterpenes and sesquiterpenes [58,59]. Based on our preliminary histomorphology results (unpublished), *P*. *amboinicus* leaves exhibited high accumulation of essential oils. Since Lamiaceae leaves produce essential oils rich in terpenoids and stems are also frequently used for essential oils extraction, both tissues were selected for *PamTps1* differential expression analysis using quantitative RT-PCR (RT-qPCR). Comparison of volatiles released by *P*. *amboinicus* leaf and stem tissues during the natural 24 h day/night cycle is displayed in S4 Fig. From this analysis, it was evident that the leaves of this herbal plant emitted a vast array of volatiles throughout the day and the amounts declined towards the night. A similar observation was made in *Lillium* whereby its floral scent emission was significantly influenced by light intensity and temperature [60].

**Fig 4:**
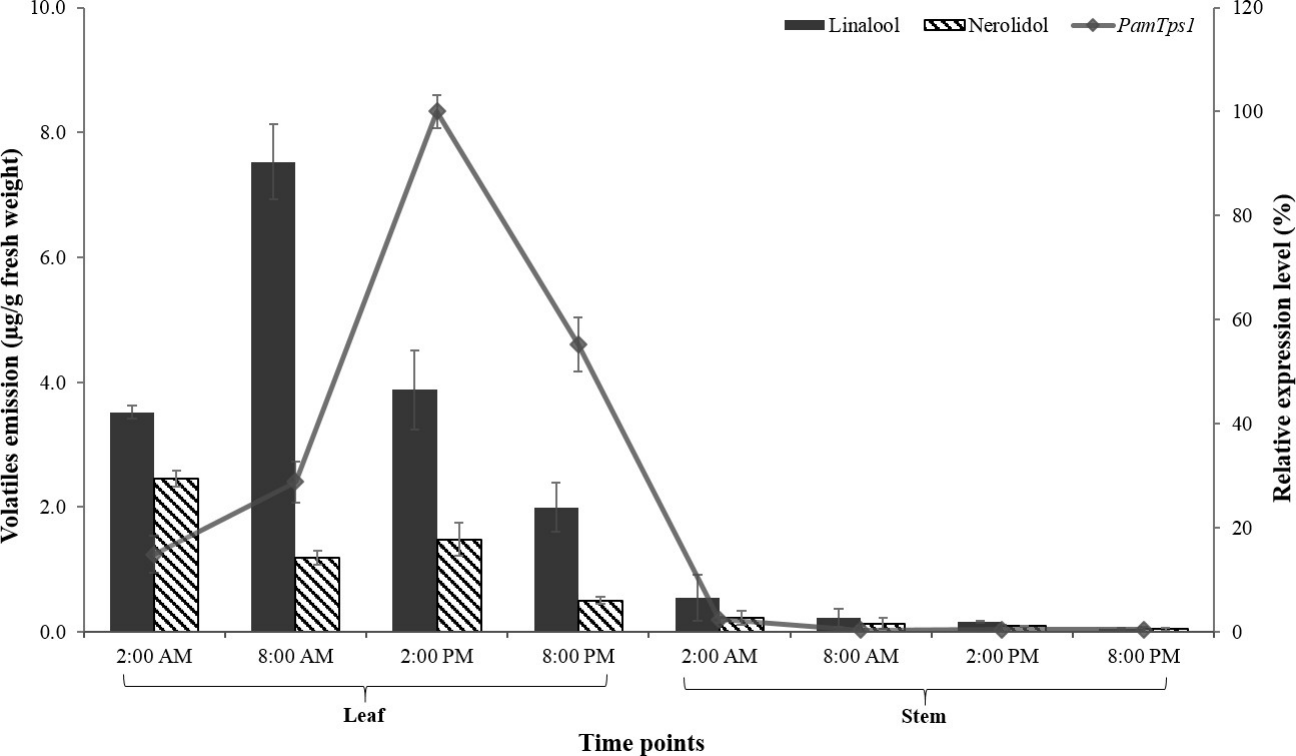
Correlation between linalool and nerolidol emissions and *PamTps1* expression in leaves and stems of *P*. *amboinicus* within a 24 h day/night cycle. Relative expression analysis was performed by qRT-PCR using *EFG*, *TUB* and *APRT* as reference genes. The relative transcription level in tissue with the highest expression quantity was set to 1 (100%). Each bar represents the mean value ±SE of three biological and three technical replicates. The linalool and nerolidol emission data presented are means ± SE of duplicate experiments.

Preferential accumulation of *PamTps*1 transcripts was observed in the leaf tissue instead of the stems, and this concurred with the emission of linalool and nerolidol from the leaves (Fig 4). Overall, our findings revealed that the *PamTps*1 showed a 42-fold preferential expression in leaves as compared to the stem with a maximal expression in the afternoon. This observation was also noted with the terpene synthases expression in *S*. *guaranitica* such as linalool synthases 1 and 2, geranyl linalool synthase, selinene synthase and β-caryophyllene synthase that showed high expression levels in the leaf tissue as compared to the stems [61]. Likewise, a *Citrus* terpene synthase exhibited preferential expression in leaves and fruit flavedo which corresponds to the terpenes accumulation and essential oils production by these tissues in the *Citrus* plants [62]. Similarly, germacrene A synthase in *Achillea millefolium* showed high expression levels in the leaves and flowers in contrast to the stem tissue [63]. In constrast, expression of linalool synthase was mostly reported in flower tissues which accounted for the floral scents emission of *Lillium* [64], *A*. *argute* [65] and *Osmanthus fragrans* [66] that correlated with the volatiles released during flower development. The expression of terpene synthase genes has been reported to be highly up-regulated in specialized cells such as those in glandular trichomes, which were located on the aerial parts of the plants [11,14,67]. A number of Lamiaceae terpene synthases, including linalool synthase, involved in terpenoid biosynthesis in secretory glandular trichomes had been functionally characterized as reviewed by Lange & Turner [68]. In *P*. *amboinicus*, our preliminary histomorphological results showed that the glandular trichomes are probably the storage sites for the essential oils produced in the leaves. However, it remains unknown whether *Pam*Tps1 expression is associated with this storage site. The *PamTps1* expression in the leaves followed a diurnal circadian rhythm with increased level exhibited early in the morning at 2.00 AM, and achieved a high point around 2.00 PM before diminishing during the remainder of the day (Fig 4). This expression pattern is similar to *A*. *chinensin* bifunctional nerolidol synthase (*AcNES1*) expression in the whole flower that showed an increment from 4.00 AM to a maximum point at midday before decreasing afterwards [69]. The *AcNES1* expression accompanied by time point analysis of terpenes suggested that nerolidol was largely accumulated throughout the day with maximal emission at midnight preceded by a steady decrease and remained low until morning. Similar trend was observed in *P*. *amboinicus* leaf where the highest nerolidol emission occurred early in the morning (2.00 AM) and the amounts reduced thereafter. In contrast, maximal linalool emission from the *P*. *amboinicus* leaf was observed at 8.00 AM and started to decline in the afternoon till midnight (Fig 4). Based on this pattern, we hypothesized that the accumulation of linalool happened during the night before the morning emission. Similar correlation was observed with Chen et al. [65] study where the *A*. *argute* linalool synthase (*AaLS1*) displayed constitutive expression with slight reduction in the morning and an increased at midday accompanied by maximal emission rate of linalool at 8.00 AM. This phenomenon could possibly be related to the study previously demonstrated in *Pinus pinea* where large emission of oxygenated monoterpenoid linalool was controlled by stomata opening, which was influenced by light intensity and temperature [70,71]. This was accompanied by a significant reduction in the emission rate of monoterpenoids during midday which was attributable to diurnal water-stress leaves with closed stomata [70]. It remains uncertain whether the diurnal water-stress leaves with closed stomata could explain the terpenoids emission pattern in *P*. *amboinicus*.

### Phylogenetic analysis of *Pam*Tps1

Phylogenetic profiling of *Pam*Tps1 protein was conducted to infer its evolutionary relationship with members of the plant terpene synthase subfamilies and other terpene synthases from the Lamiaceae family. Plant terpene synthases share a common evolutionary origin based upon their similar reaction mechanisms and conserved structural and sequence characteristics that include amino acid sequence homology and conserved sequence motifs [9,10,72]. The bifurcation of the terpene synthases involved in primary and secondary metabolism appears to occur before the separation of angiosperms and gymnosperms. Previous phylogenetic analysis of terpene synthases from the gymnosperms and angiosperms delineated the *Tps* gene family into eight subfamilies, designated as *Tpsa* through *Tpsg* and α-farnesene synthase cluster [9,72,73]. Terpene synthases that produce secondary metabolites are classified into subfamilies of *Tpsa* (angiosperm sesquiterpene and diterpene synthases), *Tpsb* (angiosperm monoterpene synthases) and *Tpsd* (gymnosperm monoterpene synthases) are only distantly related to *Tpsc* (copalyl diphosphate synthase) and *Tpse* (kaurene synthase) subfamilies that are rich in specialized (i.e. secondary) metabolism beyond carrying the enzyme involved in gibberellin biosynthesis, and distantly ancient branch *Tpsf* containing linalool synthase [9,11].

**Fig 5:**
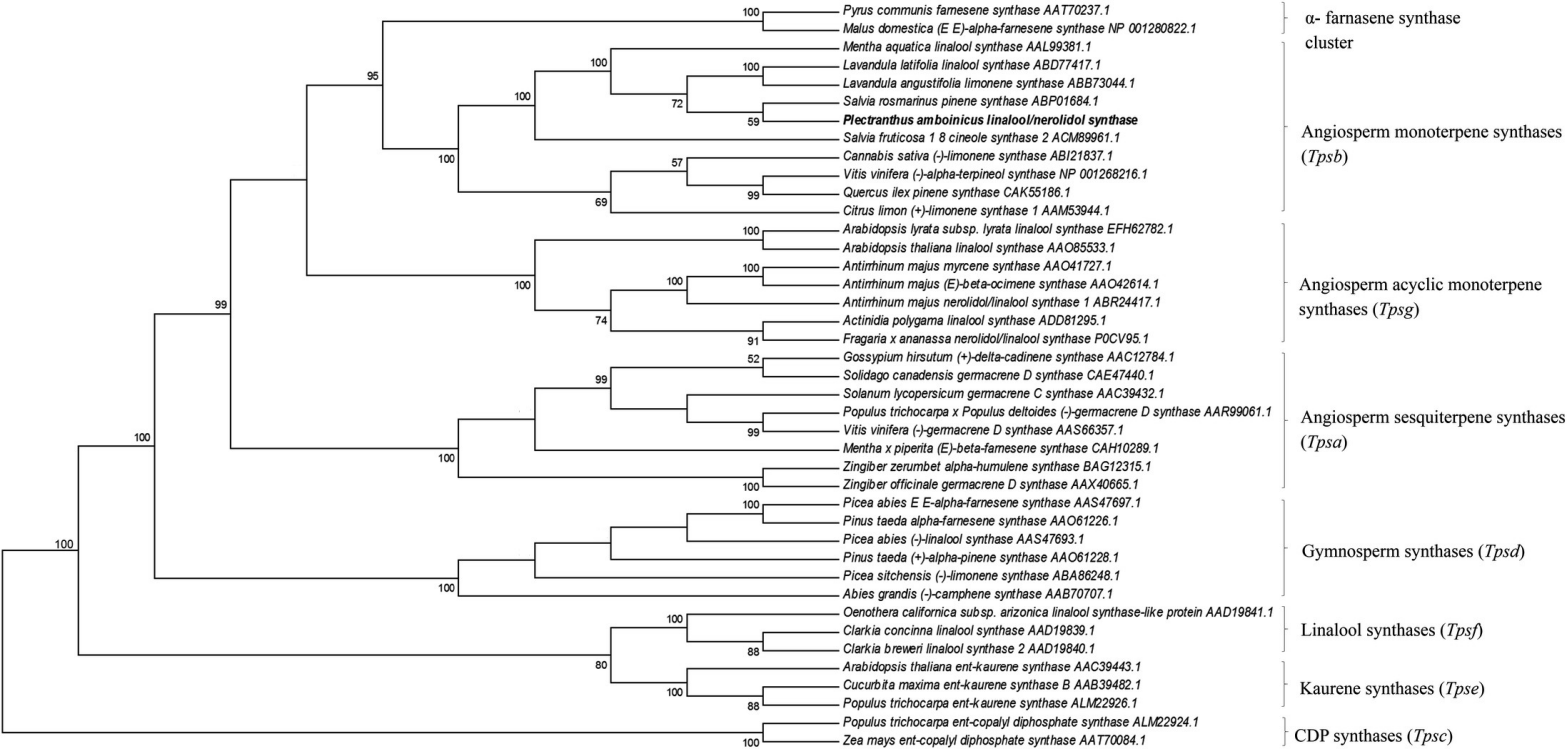
Phylogenetic relationship of *Pam*Tps1 with selected plant terpene synthases from different subfamilies. Target sequences upstream of the RRx8W motif of the alignment were removed. Selection of terpene synthases subfamilies were based on previous literatures [11,100,101]. The *Tps*c and *Tpse* subfamilies were chosen as outgroups. Evolutionary relationship was inferred using Neighbor-Joining method with 1000 replicates for bootstrapping. The numbers indicated were the actual bootstrap values of the branches.

Functional characterization of *Pam*Tps1 using *in vitro* and *in vivo* expression systems showed that this multi-substrate enzyme possesses a monoterpene synthase and a sesquiterpene synthase activity producing exclusively linalool and nerolidol, respectively. The phylogenetic tree (Fig 5) reveals that *Pam*Tps1 belongs to the *Tpsb* group as expected for a Lamiaceae monoterpene synthase. The presence of RRx_8_W motif, which is characteristic of the angiosperm *Tpsb* group [11,73], positioned *Pam*Tps1 in the *Tpsb* cluster. This monophyletic *Tpsb* group contains terpene synthases such as pinene synthase, linalool synthase and limonene synthase which produce cyclic and acyclic products, which were grouped together on the basis of their sequence similarities and the presence of tandem arginine motif, despite differences in their catalytic functions. It is evident from Fig 5 that the phylogenetically closest sequences to *Pam*Tps1 are *Lavandula* and *Salvia* terpene synthases with more than 67% similarity, signifying a close relationship between these genera that are cluster together in *Tpsb* regardless of the product specificity [67]. This could probably be related to the adaptive evolutionary process of ancestral gene copy that had undergone a divergence in structure and function which contributed to the large diversity of terpene synthases [10]. The other known multi-substrate terpene synthases that belong to the *Tpsb* and have a transit peptide are the *S*. *lycopersicum* linalool synthase [74], *Hedychium coronarium* terpene synthase [75] and β-ocimene synthase of *Arabidopsis thaliana* [45]. It is noteworthy to mention that most of the reported linalool/nerolidol synthases belong to the *Tpsg* group, a clade closely related to the *Tpsb* which comprises of angiosperm acyclic terpene synthases that produce monoterpenes, sesquiterpenes and diterpenes. However, *Tpsg* group members lack the common structural feature of RRx_8_W motif present in the *Tpsb* members [9,54].

The multi-substrate activity of terpene synthases are widely divergent across the *Tps* subfamilies in the multiple plant species. Phylogenetic analysis suggested that all plant terpene synthases originated from the ancient diterpene synthases of the *Tpsc* clade that represents the base of the rooted phylogenetic tree such as illustrated in Fig 5 [9,76,77]. These old diterpene synthases adopt a tri-domain architecture of *αβγ* proteins that contain a transit peptide [78–80]. Further evolutionary modifications over time have witnessed diversification of product profiles that are not only been associated with changes in the active site center structure, but also loss of *γ*-domain in the isoprene, monoterpene and sesquiterpene synthases, and transit peptide loss in the sesquiterpene synthases [78–80]. Identification of two plastidic and cytosolic linalool/nerolidol synthases in *A*. *majus* and *F*. *ananassa* further suggests that sesquiterpene synthases might have evolved from the monoterpene synthases ancestor through the loss of plastid signal peptide and acquirement of an active site adaptation to the FPP substrate [46,48,81,82]. The evolution of terpene synthases family shows that these enzymes possesses a remarkable flexibility to evolve into new functional diversification and subsequently contributes to the chemical diversity of terpenoids in nature [9].

## Conclusions

Initially, *Pam*Tps1 was selected as a terpene synthase candidate among other candidates for functional characterization study based on the highest sequence similarity with available plant terpene synthases in the database. The functional study has undoubtedly classified *Pam*Tps1 as a linalool/nerolidol synthase with the ability to exclusively produce linalool and nerolidol in both *in vivo* and *in vitro* systems, suggesting that this enzyme possesses both monoterpene synthase and sesquiterpene synthase activities. Interestingly, *PamT*ps1 was clustered with the *Tpsb* subfamily as expected for a predicted monoterpene synthase instead of *Tpsg* or *Tpsf* which was presumed for an acyclic monoterpene synthase or ancient linalool synthase, respectively. Although this study did not clarify the exact role of *Pam*Tps1 in *P*. *amboinicus*, it did not rule out the possibility that *Pam*Tps1 could function as both a monoterpene synthase and a sesquiterpene synthase *in planta*. The expressional analysis showed that this transcript was highly expressed in *P*. *amboinicus* leaves throughout the day that correlated with its linalool emission following a diurnal circadian pattern. Even though *Pam*Tps1 is only accountable for production of minor volatiles in *P*. *amboinicus*, it represents the first substrate promiscuous monoterpene synthase that has been cloned and functionally characterized from this herbal plant. The substrate promiscuity activity of *Pam*Tps1 has intrigued us to further study this enzyme for the opportunity to attain additional insight into its catalytic basis of product specificity.

## Supporting information

S1 Table qPCR primers used in this study. (PDF)

S1 Fig SDS-PAGE and Western blot profile of purified *Pam*Tps1. Lane M: Full-Range Rainbow™ Molecular Weight Markers (GE Healthcare, USA). Lanes 1–3: *Pam*Tps1 protein from IMAC fractions and lane 4: *Pam*Tps1 from gel filtration fraction on SDS-PAGE analysis (A) and Western blot analysis (B). The recombinant *Pam*Tps1 band was observed with the expected size of 79 kDa.
(TIF)

S2 Fig Mass spectra comparison of products generated by recombinant *Pam*Tps1. Mass spectra of linalool (A) and (B) nerolidol generated by *Pam*Tps1; mass spectra of authentic (-)-linalool (C) and *trans*-nerolidol (D) standards; mass spectra of linalool (E) and *trans*-nerolidol (F) in the NIST14 library.
(TIF)

S3 Fig GC-MS chromatograms (selected ion, m/z = 93) negative control reactions of *in vitro* assay. Negative control reaction without presence of *Pam*Tps1 (A) and empty vector control reaction (B) after incubation with GPP; negative control reaction without presence of *Pam*Tps1 (C) and empty vector control reaction (D) after incubation with FPP. Corresponding compounds are: 1 = linalool (retention time = 6.7 min); 2 = *trans*-nerolidol (retention time = 13.0 min) and 3 = *cis*–nerolidol (retention time = 12.6 min).
(TIF)

S4 Fig Comparison of chromatographic profiles of volatiles emitted from different tissues of *P*. *amboinicus* within a 24 h day/night cycle. TIC profiles of (A) leaf and (B) stem at 2.00 AM; (C) leaf and (D) stem at 8.00 AM; (E) leaf and (F) stem at 2.00 PM; and (G) leaf and (H) stem at 8.00 PM. Corresponding retention time for linalool and *trans*–nerolidol are 13.7 min and 26.0 min, respectively.
(TIF)

S1 Raw images (PDF)
