# Supplementary material for: Functional characterization of a new terpene synthase from Plectranthus amboinicus
Source: PLoS One. 2020 Jul 2;15(7):e0235416. doi: 10.1371/journal.pone.0235416 (PMC7332032; doi:10.1371/journal.pone.0235416)
Supplement: S1 Table — (PDF) [file pone.0235416.s001.pdf]

**Genes**	**Sequences**	**T_m (°C)**	**Amplicon size (bp)**
PamTps1	F - GGAACAGCACCCTTTGAGC	63.80	291
	R - GAACAGCAACTCCGCCATC	63.89	
APRT	F - TCCCACCCCTCAAAATCTGC	59.96	222
	R - CGTCAGCATTGGAAGTTGCC	60.11	
TUB	F - GGCTCTGGTTTGGGGTCTTT	60.18	233
	R - GTGGGCCTCTCAATGTCCAA	59.96	
EFG	F - GCCAGTTCGCCGATATCACT	60.25	178
	R- CGAACGTCAACCACAGGGTA	59.97	
