# Supplementary figures and images for: Functional characterization of a new terpene synthase from Plectranthus amboinicus
Source: PLoS One. 2020 Jul 2;15(7):e0235416. doi: 10.1371/journal.pone.0235416 (PMC7332032; doi:10.1371/journal.pone.0235416)

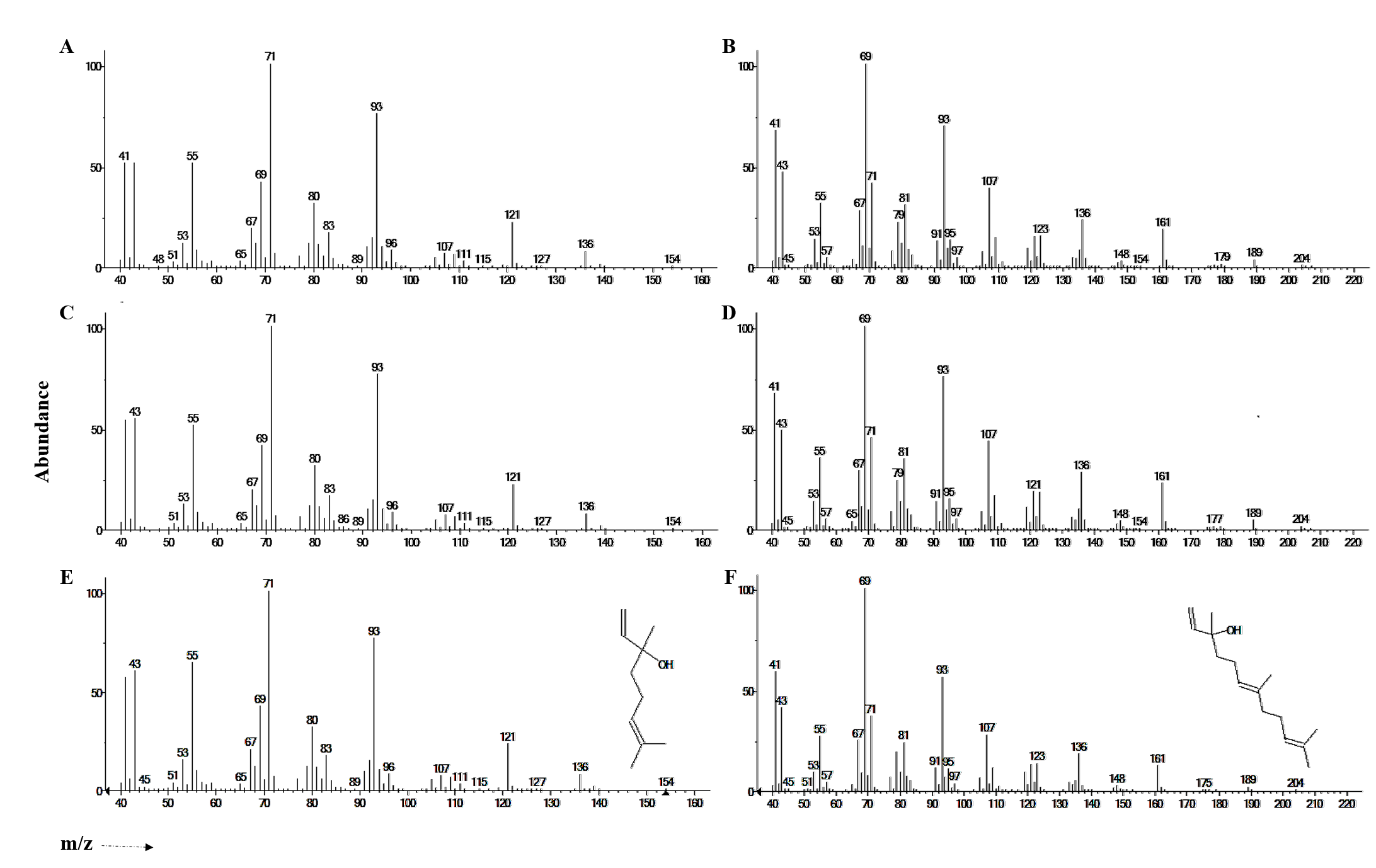

Supplement: S2 Fig — Mass spectra of linalool (A) and (B) nerolidol generated by PamTps1; mass spectra of authentic (-)-linalool (C) and trans-nerolidol (D) standards; mass spectra of linalool (E) and trans-nerolidol (F) in the NIST14 library. (TIF) [file pone.0235416.s003.tif]

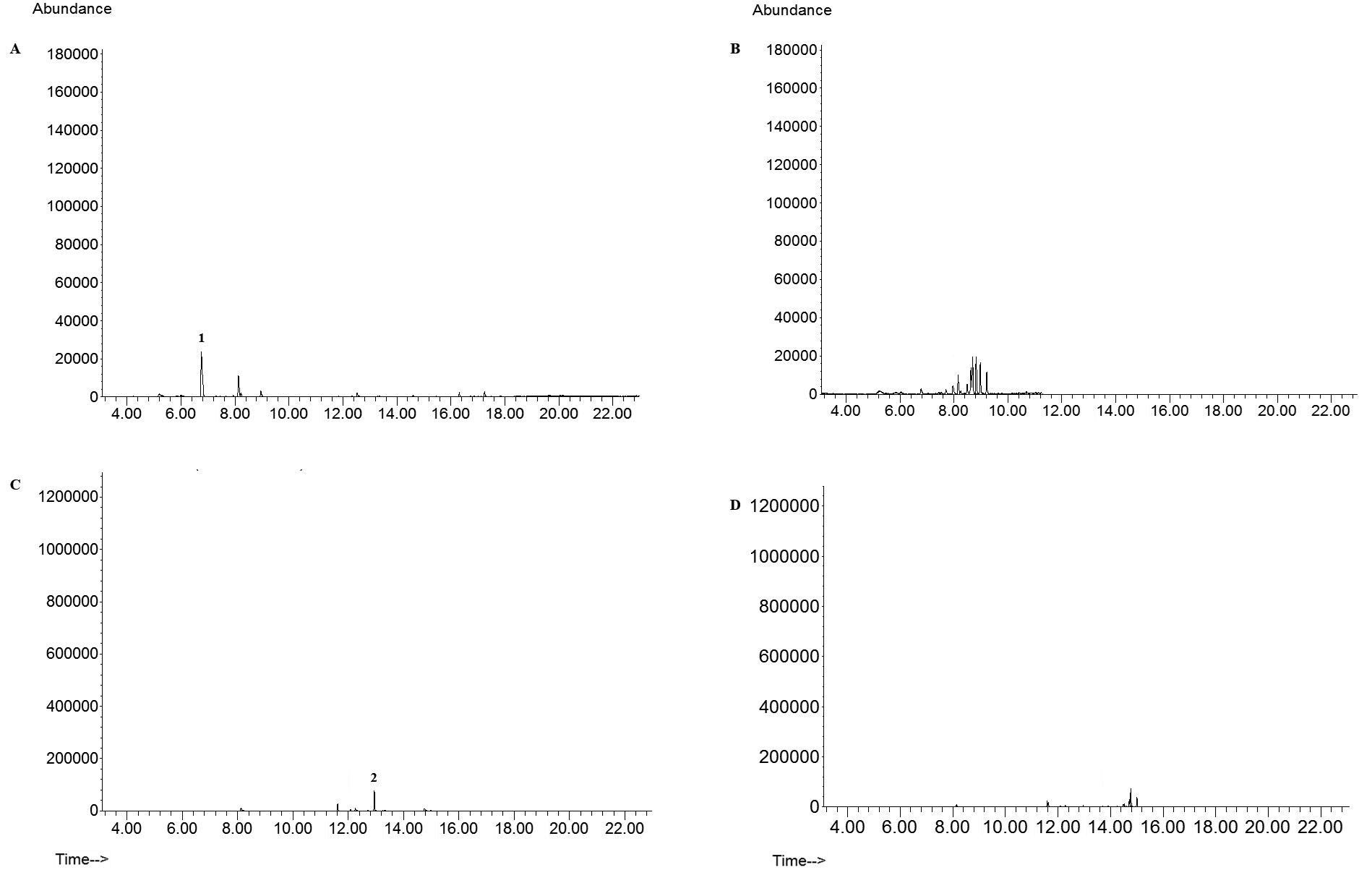

Supplement: S3 Fig — Negative control reaction without presence of PamTps1 (A) and empty vector control reaction (B) after incubation with GPP; negative control reaction without presence of PamTps1 (C) and empty vector control reaction (D) after incubation with FPP. Corresponding compounds are: 1 = linalool (retention time = 6.7 min); 2 = trans-nerolidol (retention time = 13.0 min) and 3 = cis–nerolidol (retention time = 12.6 min). (TIF) [file pone.0235416.s004.tif]

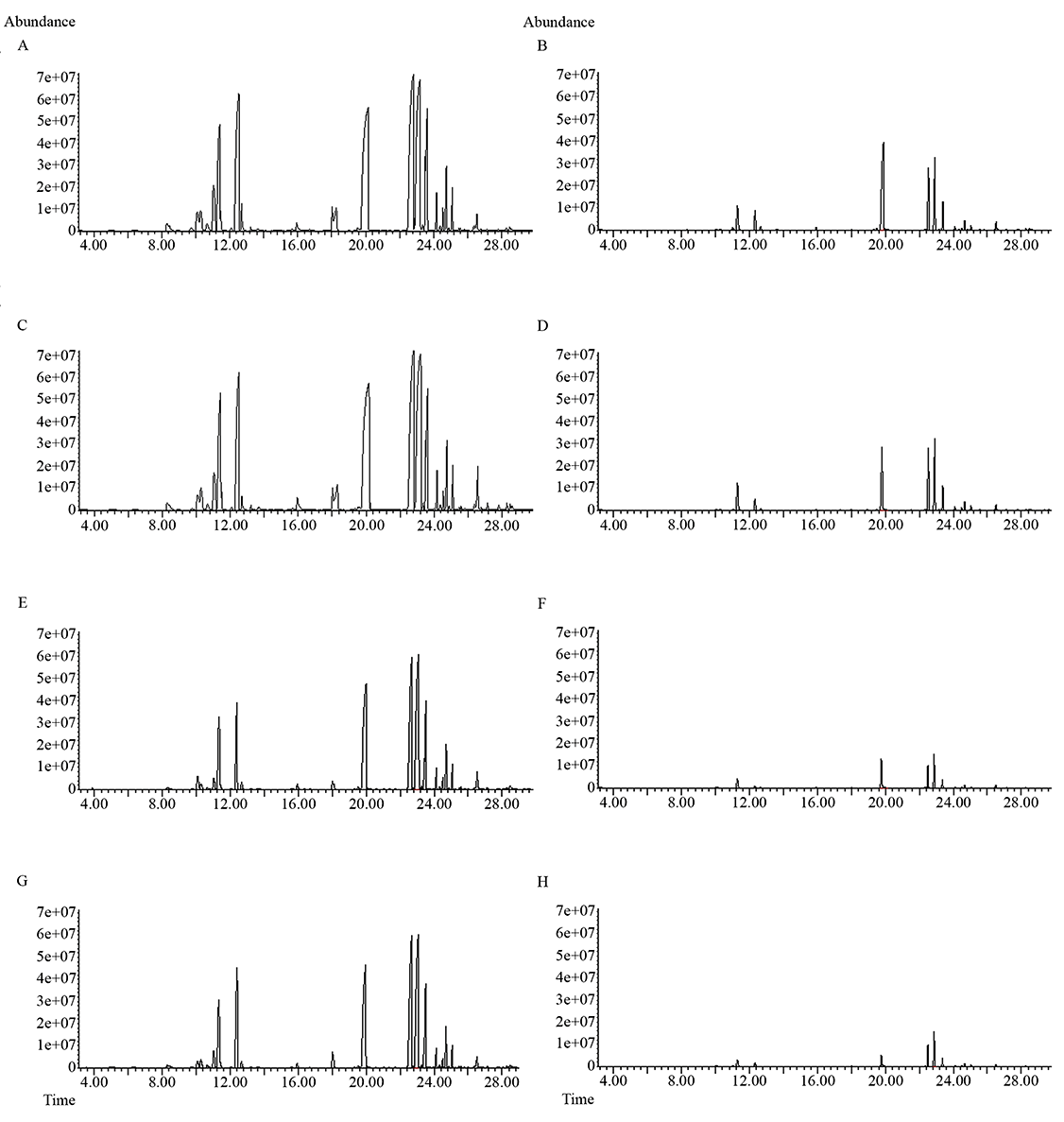

Supplement: S4 Fig — TIC profiles of (A) leaf and (B) stem at 2.00 AM; (C) leaf and (D) stem at 8.00 AM; (E) leaf and (F) stem at 2.00 PM; and (G) leaf and (H) stem at 8.00 PM. Corresponding retention time for linalool and trans–nerolidol are 13.7 min and 26.0 min, respectively. (TIF) [file pone.0235416.s005.tif]
