# Supplementary material for: Functional characterization of a new terpene synthase from Plectranthus amboinicus
Source: PLoS One. 2020 Jul 2;15(7):e0235416. doi: 10.1371/journal.pone.0235416 (PMC7332032; doi:10.1371/journal.pone.0235416)
Supplement: S1 Raw images — (PDF) [file pone.0235416.s006.pdf]

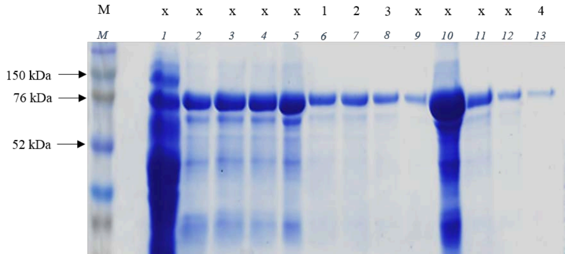
M x x x x x 1 2 3 x x x x 4
M 1 2 3 4 5 6 7 8 9 10 11 12 13
150 kDa
76 kDa
52 kDa

Raw S1 Fig for SDS-PAGE profile

SDS-PAGE profile of *Pam*Tps1 two-steps purification. Lane M: Full-Range Rainbow™ molecular weight markers; lane 1: crude extract; lanes 2-9: IMAC fractions; lane 10: pooled and concentrated IMAC fractions; lanes 11-13: GF fractions.

Lanes label with x represent lanes not included in the final S1 Fig. The original lanes of M, *6-8* and *13* represent lanes M, 1-3 and 4 in the final S1 Fig, respectively.

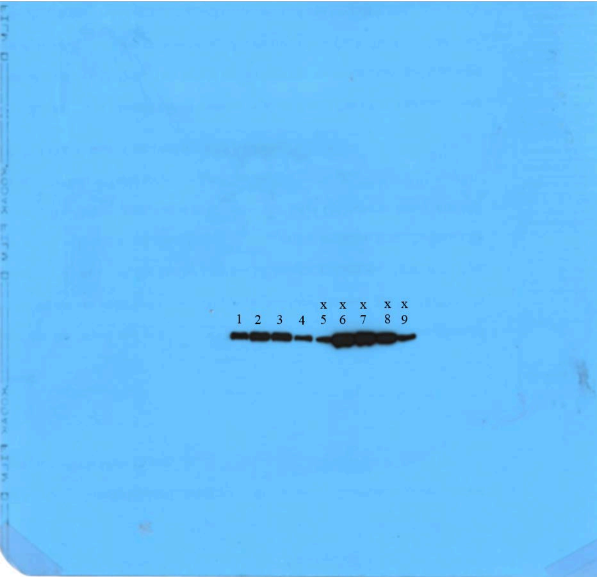
x x x x x
1 2 3 4 5 6 7 8 9

<u>Raw S1 Fig for Western blot profile</u>

Raw Western blot profile of *Pam*Tps1. Lanes 1-3: *Pam*Tps1 protein from IMAC fractions; lane 4: *Pam*Tps1 from gel filtration fraction; lane 5: crude extract; lane 6: Total cell protein (TCP) induced at 24 h, lane 7: TCP induced at 6 h; lane 8: TCP induced at 4 h and lane 9: TCP induced at 2 h.

Lanes label with x represent lanes not included in the final S1 Fig. The lanes label as 1-4 are included in the final S1 Fig in the same arrangement.
